# Targeting Tumor Cells Overexpressing the Human Epidermal Growth Factor Receptor 3 with Potent Drug Conjugates Based on Affibody Molecules

**DOI:** 10.3390/biomedicines10061293

**Published:** 2022-05-31

**Authors:** Sara S. Rinne, Wen Yin, Anna Mestre Borras, Ayman Abouzayed, Charles Dahlsson Leitao, Anzhelika Vorobyeva, John Löfblom, Stefan Ståhl, Anna Orlova, Torbjörn Gräslund

**Affiliations:** 1 Department of Medicinal Chemistry, Uppsala University, 751 23 Uppsala, Sweden; sara.rinne@ilk.uu.se (S.S.R.); ayman.abouzayed@ilk.uu.se (A.A.); 2 Department of Protein Science, KTH Royal Institute of Technology, 114 17 Stockholm, Sweden; wenyin@kth.se (W.Y.); annamb@kth.se (A.M.B.); chdl@kth.se (C.D.L.); lofblom@kth.se (J.L.); ssta@kth.se (S.S.); 3 Department of Immunology, Genetics and Pathology, Uppsala University, 752 37 Uppsala, Sweden; anzhelika.vorobyeva@igp.uu.se; 4 Science for Life Laboratory, Uppsala University, 752 37 Uppsala, Sweden

**Keywords:** affibody molecule, human epidermal growth factor receptor 3 (HER3), BxPC-3, emtansine, DM1, albumin binding domain, affibody drug conjugate (AffiDC)

## Abstract

Increasing evidence suggests that therapy targeting the human epidermal growth factor receptor 3 (HER3) could be a viable route for targeted cancer therapy. Here, we studied a novel drug conjugate, Z_HER3_-ABD-mcDM1, consisting of a HER3-targeting affibody molecule, coupled to the cytotoxic tubulin polymerization inhibitor DM1, and an albumin-binding domain for in vivo half-life extension. Z_HER3_-ABD-mcDM1 showed a strong affinity to the extracellular domain of HER3 (K_D_ 6 nM), and an even stronger affinity (K_D_ 0.2 nM) to the HER3-overexpressing pancreatic carcinoma cell line, BxPC-3. The drug conjugate showed a potent cytotoxic effect on BxPC-3 cells with an IC_50_ value of 7 nM. Evaluation of a radiolabeled version, [^99m^Tc]Tc-Z_HER3_-ABD-mcDM1, showed a relatively high rate of internalization, with a 27% internalized fraction after 8 h. Further in vivo evaluation showed that it could target BxPC-3 (pancreatic carcinoma) and DU145 (prostate carcinoma) xenografts in mice, with an uptake peaking at 6.3 ± 0.4% IA/g at 6 h post-injection for the BxPC-3 xenografts. The general biodistribution showed uptake in the liver, lung, salivary gland, stomach, and small intestine, organs known to express murine ErbB3 naturally. The results from the study show that Z_HER3_-ABD-mcDM1 is a highly potent and selective drug conjugate with the ability to specifically target HER3 overexpressing cells. Further pre-clinical and clinical development is discussed.

## 1. Introduction

Approaches to specifically target receptors that are abnormally expressed on cancer cells have become a viable therapeutic strategy and several drugs are in clinical trials or have been approved for clinical use. One of the most common targeting moieties are monoclonal antibodies (mAbs) [1]. However, pre-clinical as well as clinical trials have also shown that fragments of mAbs [2] or engineered affinity scaffold proteins (EAPs) [3], may be used.

A well-studied group of receptors with relevance to cancer is the epidermal growth factor receptor (ERBB) family, for which many targeted therapies have been investigated. The members of the ERBB family are tyrosine kinase receptors, which are involved in driving tumor progression and in the acquisition of resistance to therapy [4]. One of the family members, the human epidermal growth factor receptor 3 (HER3, human ErbB3), is a transmembrane receptor with impaired tyrosine kinase activity. It has been observed that the expression level of HER3 is associated with malignancy for several different cancer types, such as non-small cell lung carcinoma (NSCLC) [5], pancreatic carcinoma [6], melanoma [7], prostate carcinoma [8], and breast carcinoma [9]. The two main ligands of HER3 are heregulin (HRG) and neuregulin 2 (NRG2). Upon binding, HRG and NRG2 activate the receptor, which in turn can form heterodimers with the other members of the ERBB family, followed by the activation of downstream signaling [10]. HRG and NRG2 expression is sometimes upregulated, which over-activates HER3, which in turn drives tumor growth [11].

Due to HER3’s upregulated expression in different cancers, therapies specifically targeting HER3 are of increasing interest. Seribantumab is a mAb which has shown promise in combination with other drugs in clinical phase I or II studies for several different indications, including breast carcinoma, NSCLC, colorectal carcinoma, and ovarian carcinoma [4]. Other HER3-targeting mAbs that have shown promise in early clinical trials include patritumab, lumretuzumab, and elgemtumab.

A more recent approach to HER3-targeted therapy is to link the mAb to a potent cytotoxic drug, resulting in an antibody drug conjugate (ADC) [12,13]. These types of targeted drugs often have several modes of action: (i) the mAb part may prevent the transduction of signaling by the targeted receptor, and/or induce an antibody-dependent cellular cytotoxicity (ADCC) response, (ii) the linked drug delivers a cytotoxic effect leading to cell death. Until now, eleven ADCs have been approved for clinical use for different cancer indications by the regulatory authorities in Europe and/or the USA. Data from studies of these compounds suggest that they are often well-tolerated and efficient therapy options. However, severe side-effects occur for some patients; most commonly liver damage from off-target uptake of the ADC, and a low blood count from premature release of the cytotoxic drug from the mAb [14]. Patritumab deruxtecan is a HER3-targeting ADC in phase I/II clinical trials for patients suffering from NSCLC and breast carcinoma, and has shown promising results [15]. Compared to other ADCs, patritumab deruxtecan has a relatively weak affinity for its receptor and a relatively high rate of internalization [16].

Another drug candidate under clinical development is zenocutuzumab, a bi-specific mAb targeting HER2 (human epidermal growth factor receptor 2) and HER3. Zenocutuzumab prevents HRG binding to HER3 and has been engineered for an enhanced ADCC response. It was granted fast track designation by the U.S. Food and Drug Administration (FDA) in early 2021 for the clinical testing of metastatic, HRG-positive solid tumors. Three patients, one with NSCLC and two with pancreatic carcinoma receiving zenocutuzumab all responded with tumor shrinkage [17].

The early clinical evaluations of HER3-targeted therapies on small cohorts have shown promise. However, the few clinical phase III studies performed on mAbs have thus far not been successful [4]. Furthermore, clinical phase III studies on HER3-targeting ADCs have not yet been undertaken. While HER3 targeting may be a viable strategy for cancer treatment, based on the clinical evaluations presented above, it is not evident how to best design a HER3-targeting drug. Compared to many other plasma membrane-anchored receptors, for which targeted therapies have been developed, HER3 has a substantial expression on normal tissues, and its overexpression in tumors is only modest for most patients, up to approximately 50,000 receptors/tumor cells [18], which makes the development of targeted therapies challenging.

Affibody molecules are engineered affinity scaffold proteins (EAPs) derived from the B-domain of protein A from *Staphylococcus aureus*, normally folding into an anti-parallel triple helical structure [19]. They consist of 58 amino acids (Mw 7 kDa) and the scaffold is devoid of cysteine amino acids. The scaffold has a natural affinity for some IgGs. By randomizing 13 amino acids in the two helices directly involved in IgG binding or in close vicinity to the binding surface, combinatorial libraries have been generated. From these libraries, variants that no longer bind to IgG but to desired targets have been selected by e.g., phage or cell display techniques [20].

Affibody molecules, specifically interacting with HER3 have been described [21,22]. These binders have been radiolabeled and investigated as radionuclide molecular imaging agents in pre-clinical mouse models, where they were able to visualize HER3-expressing tumors and to discriminate between high and low receptor expression [23,24,25,26,27,28,29]. The most promising variant for molecular imaging was Z_HER3:08698_ with specific and strong interaction with HER3 (equilibrium dissociation constant, K_D_ 50 pM) [22]. When evaluated for imaging, a tag with the amino acid sequence His-Glu-His-Glu-His-Glu, a (HE)_3_-tag, was placed in the N-terminus. It was used for purification by immobilized metal-ion affinity chromatography (IMAC), and for radiolabeling with [^99m^Tc]Tc. It has also been shown to minimize unspecific uptake in the liver compared to the more commonly used hexahistidine tag [30,31,32]. We hypothesized that affibody molecules binding to HER3 could be used to create drug conjugates, similar to an antibody drug conjugate.

To create an affibody drug conjugate (AffiDC), a suitable payload has to be conjugated with the affibody carrier. DM1 is a highly toxic tubulin polymerization inhibitor derived from maytansine [33]. It is for example used in trastuzumab emtansine (T-DM1), an FDA-approved ADC for the treatment of HER2-positive, metastatic breast cancer [34]. In T-DM1, the payload is linked via a non-cleavable linker. These types of ADCs bind to their intended receptor on the tumor cells, become internalized, and are transported to the lysosome where the protein part is degraded. When the hydrophobic drug is separated from the protein part, it can enter the cytosol by diffusion through the lysosomal membrane and can inhibit tubulin polymerization, leading to cell death.

Affibody molecules are small and are quickly excreted from circulation due to renal filtration. Different methods can be employed to extend the in vivo half-life [35]. One strategy often used for affibody molecules is head-to-tail fusion with an albumin-binding domain (ABD), which upon entering the blood stream associates with albumin, leading to an increase in the molecular weight of the complex by the molecular weight of albumin (67 kDa). These complexes become larger than the cut-off of renal filtration, extending the plasma half-life [36]. In particular, ABD_035_, with sub-picomolar affinity (K_D_) has been found to be suitable as a fusion partner to affibody molecules [37]. A potential drawback with the use of an albumin-binding domain in cancer drugs is that some cancer patients develop hypoalbuminemia. It occurs, for example, in approximately 40% of all patients with pancreatic carcinoma [38], in which cases the bioavailabilty of ABD-containing drugs may be lower than for patients with a normal albumin level.

We have previously investigated AffiDCs targeting HER2, a receptor belonging to the same family as HER3 [36,39,40,41]. From these studies it was found that an architecture consisting of a monovalent affibody-targeting domain followed by an ABD led to the best performing AffiDC. Furthermore, a C-terminal extension after the ABD, with the amino acid sequence Glu-Glu-Glu-Cys, where the cytotoxic DM1 was attached to the cysteine, was found to minimize unwanted unspecific uptake in the liver [39,40].

In this study, we generated a novel drug conjugate targeting HER3, based on the affibody molecule (Z_HER3:08698_) carrying the drug DM1. We utilized an analogous architecture to the best performing HER2-targeting AffiDC [40]. The drug conjugate was characterized biochemically, and its cytotoxic potential was evaluated. After radiolabeling with [^99m^Tc]Tc, the specificity of binding and rate of internalization was determined, and the distribution in mice bearing HER3-positive xenografts was quantified.

## 2. Materials and Methods

### 2.1. General

Unless stated otherwise, all chemicals were from Merck (Darmstadt, Germany) or Sigma-Aldrich (St. Louis, MO, USA). Restriction digestion enzymes were from New England Biolabs (Ipswitch, MA, USA). Statistical significance (*p <* 0.05) was determined using Prism, version 9.3.1 (Graphpad Software, San Diego, CA, USA). For comparison of two groups, a Mann–Whitney test was used. For comparison of multiple groups, a Kruskal–Wallis test with a post-hoc Dunn’s test was used.

### 2.2. Gene Construction

The HER3-targeting affibody molecule used in this study was Z_HER3:08698_ [22], herein abbreviated as Z_HER3_. The albumin-binding domain used for in vivo half-life extension was ABD_035_ [37], herein abbreviated as ABD. Starting from a plasmid-encoding Z_HER3_-ABD [29], the affibody coding gene was sub-cloned to the pET-21a(+) plasmid (Novagen, Madison, WI, USA) by in-fusion cloning according to the manufacturer’s recommendations (Takara, Kusatsu, Japan). The sequence consisted of codons encoding a Met-His-Glu-His-Glu-His-Glu tag in the N-terminus, followed by the Z_HER3_ affibody sequence, the sequence encoding the ABD domain, and codons encoding a Glu-Glu-Glu-Cys extension in the C-terminus. The plasmid was sequenced by sanger sequencing (Eurofins genomics, Ebersberg, Germany).

### 2.3. Protein Expression and Purification

The plasmid-encoding Z_HER3_-ABD was transformed to *Escherichia coli* BL21 (DE3) cells (Thermo Fisher Scientific, Waltham, MA, USA) for protein production. Transformed cells were grown in 1 L cultures in TSB-medium (30 g/L tryptic soy broth, 5 g/L yeast extract and 50 μg/L Kanamycin), in 5 L shake flasks, at 200 rpm (37 °C). When the OD_600_ was approximately 0.9, isopropyl-β-D-1-thiogalactopyranoside (Appolo Scientific, Stockport, UK) was supplemented (1 mM final concentration) to induce protein expression. The cells were then grown for 16 h at 25 °C, after which they were collected by centrifugation. The cells were lysed by sonication and the proteins were purified by affinity chromatography using the ligand human serum albumin (HSA) immobilized on a sepharose column on an ÄKTA pure system (GE Healthcare, Uppsala, Sweden). TST (25 mM Tris-HCl, 1 mM EDTA, 200 mM NaCl, 0.05% Tween, pH 8.0) was used as a running buffer, ammonium acetate (5 mM, pH 5.5) was used for washing, and acetic acid (0.5 M, pH 2.8) was used for elution. The protein were subsequently lyophilized.

### 2.4. Conjugation with DM1

The lyophilized protein was reconstituted in PBS (pH 6.7) to 0.1 mM as the final concentration. To reduce potentially oxidized cysteines, freshly prepared tris (2-carboxyethyl) phosphine (TCEP, pH 6.7) was added to 5 mM as the final concentration, followed by incubation at 37 °C for 1 h. Maleimidocaproyl-DM1 (mcDM1) (Levena Biopharma, San Diego, CA, USA) was added to 0.3 mM as the final concentration, followed by overnight incubation at room temperature. Unreacted mcDM1 was removed by passage through a NAP-5 desalting column.

Reversed-phase high-performance liquid chromatography (RP-HPLC) was used for further purification. Eluted material from the NAP-5 column was loaded on a Zorbax SB-C18 semi-preparative column (Agilent, Santa Clara, CA, USA), followed by elution with a gradient from 25% to 65% acetonitrile in water, supplemented with 0.1% trifluoroacetic acid (TFA), for 40 min. The fractions containing Z_HER3_-ABD-mcDM1 were pooled and lyophilized.

A non-toxic control construct, Z_HER3_-ABD-AA, was also created, where the C-terminal cysteine was alkylated. Lyophilized Z_HER3_-ABD was reconstituted in a 200 mM ammonium bicarbonate buffer (pH 8.0) supplemented with 2% sodium dodecyl sulfate. Freshly dissolved TCEP was added to 10 mM as the final concentration, followed by incubation for 1 h at 55 °C. Freshly prepared 2-iodoacetamide was added to 20 mM as the final concentration, followed by incubation for 30 min in the dark. Z_HER3_-ABD-AA was purified by RP-HPLC and lyophilized in the same way as Z_HER3_-ABD-mcDM1.

### 2.5. Biochemical Characterization

The two constructs, Z_HER3_-ABD-mcDM1 and Z_HER3_-ABD-AA, were reconstituted in PBS, and were characterized biochemically by sodium dodecyl sulphate-polyacrylamide gel electrophoresis (SDS-PAGE), analytical size-exclusion chromatography (SEC), analytical RP-HPLC, and circular dichroism spectroscopy (CD).

For the SDS-PAGE analysis, 10 μg of each construct was loaded on a NuPAGE Bis-Tris protein gel. After 1 h electrophoresis at 200 V, the gel was stained with Gelcode blue safe protein stain for 1 h. Then, the gel was destained with water and photographed.

For analytical SEC, 5 μg protein was analyzed on a prepacked Superdex-75 5/150 column (GE Healthcare). The column was equilibrated with PBS and the samples were loaded followed by elution with PBS at a flow rate of 0.45 mL/min.

For analytical RP-HPLC, the constructs were loaded on a Zorbax 300SB-C18 column (Agilent), followed by elution by a 25 min linear gradient from 30% to 60% acetonitrile in water supplemented with 0.1% TFA.

Circular dichroism (CD) measurements were carried out on a Chirascan spectropolarimeter (Applied Photophysics, Leatherhead, UK). A cuvette with an optical path length of 1 mm was used. To determine thermal stability, a sample (0.4 mg/mL) of each construct was heated from 20 to 95 °C (5 °C/min). During the heating process, the ellipticity at 221 nm was measured. Since both Z_HER3_ and ABD have a high alpha-helical content, the ellipticity at 221 nm is an estimate of helical unfolding and can be used for melting temperature (T_m_) estimation. Spectra before and after heating to 95 °C were recorded between 195 and 260 nm.

### 2.6. Affinity Determination

The affinities of the affibody constructs towards HER3 and murine ErbB3 were measured by surface plasmon resonance analysis using a capture setup on a Biacore 8K instrument (GE Healthcare). Human serum albumin (HSA) was immobilized on a CM5 chip using an amine coupling kit, according to the manufacturer’s protocol (GE Healthcare). The level of HSA immobilization in each channel was approximately 1000 RU. The affibody constructs were captured on the surface by a 30 s injection of a 100 nM solution. Finally, a multi-cycle kinetic analysis with a dilution series of HER3 or murine ErbB3 (6.3 nM, 12.5 nM, 25 nM, 50 nM, and 100 nM) was performed at two different temperatures, 25 °C and 37 °C. Samples heated to 95 °C and then cooled down to 25 °C during CD measurements were also analyzed in the same way. The flow rate during the experiments was 30 μL/min. The chip surfaces were regenerated between the runs by injecting 10 mM HCl during 30 s. The sensorgrams were analyzed using a Langmuir 1:1 kinetic model.

### 2.7. Cell Lines

The BxPC-3 (pancreatic cancer) and DU145 (prostate cancer) cell lines were purchased from ATCC (Manassas, VA, USA) and were maintained in an RPMI 1640 growth medium supplemented with 10% fetal bovine serum (FBS) (Sigma-Aldrich, St. Louis, MO, USA). A Trypsin-EDTA solution (Sigma-Aldrich) was used for cell detachment. The cells were grown in a humidified incubator at 37 °C in a 5% CO_2_ atmosphere.

### 2.8. Determination of Cytotoxicity

To investigate the cytotoxic potential of Z_HER3_-ABD-mcDM1, 5000 BxPC-3 cells were seeded per well in 96-well plates and were allowed to attach for 24 h. Then, the medium was aspirated, and a new medium was added containing a series of concentrations of Z_HER3_-ABD-mcDM1 or Z_HER3_-ABD-AA. The cells were incubated for 72 h, and then cell viability was determined with the Cell Counting Kit-8 (Sigma-Aldrich).

### 2.9. Radiolabeling with [^99m^Tc]Tc and Analysis of the Stability of the Labeled Constructs

Both Z_HER3_-ABD-mcDM1 and Z_HER3_-ABD-AA were radiolabeled with [^99m^Tc]Tc(CO)_3_ on the N-terminal amino acid sequence His-Glu-His-Glu-His-Glu according to a previously published protocol [25,42]. Briefly, [^99m^Tc]NaTcO_4_ eluate (400–500 μL, ~4 GBq) was added to a glass vial containing a CRS kit (PSI, Villigen, Switzerland) and was incubated at 100 °C for 30 min. Thereafter, 40 µL (340–400 MBq) of the obtained [^99m^Tc]Tc(CO)_3_ solution was added to 50 µg of the affibody constructs in 40 μL PBS. The mixture was incubated at 50 °C for 60 min.

The radiochemical yield was determined by Instant Thin Layered Chromatography (ITLC). A sample of the reaction mixture was applied to a Silica Gel impregnated Chromatography strip (Agilent) and was eluted with PBS. To check for the presence of reduced hydrolyzed technetium, a sample was applied to a second strip, and was eluted with pyridine:acetic acid:water (5:3:1.5).

The radiolabeled conjugates were purified using NAP-5 size-exclusion columns (GE Healthcare), pre-equilibrated with 1% BSA in PBS, and the purity was determined by ITLC. To test the stability of the technetium label, 1 µg each of [^99m^Tc]Tc-Z_HER3_-ABD-mcDM1 and [^99m^Tc]Tc-Z_HER3_-ABD-AA were incubated with a 500-fold molar excess of histidine at room temperature and at 37 °C. Control samples in PBS were also incubated at room temperature for up to 4 h. The release of the radiolabel was determined by ITLC.

A control for the biodistribution experiment, (HE)_3_-Z_HER3_, was similarly labeled according to a previously published protocol [25].

### 2.10. Binding Specificity, Internalization, and Retention

To confirm the binding specificity of [^99m^Tc]Tc-Z_HER3_-ABD-mcDM1 and [^99m^Tc]Tc-Z_HER3_-ABD-AA, HER3-expressing BxPC-3 and DU145 cells were incubated with 0.1 nM of the radiolabeled constructs for 1 h at 37 °C. Prior to incubation, HER3 receptors were pre-saturated by the addition of 50 nM of the Z_HER3:0698_ affibody molecule. After incubation, the cells were detached, collected, and measured for radioactivity content.

Internalization of [^99m^Tc]Tc-Z_HER3_-ABD-mcDM1 was studied in BxPC-3 and DU145 cells using the ‘acid wash’ method [24]. In brief, the cells were incubated with 0.1 nM [^99m^Tc]Tc-Z_HER3_-ABD-mcDM1 at 37 °C for 1, 2, 4, and 8 h. At each time point a set of dishes was removed from the incubator and cells were incubated with 0.2 M glycine buffer (with 0.15 M NaCl, 4 M Urea, pH 2.0) for 5 min on ice. The solution was collected and considered membrane-bound activity. Thereafter the cells were incubated with 1 M NaOH for 30 min at 37 °C, scraped, and collected. The collected activity from these samples was considered to be the internalized fraction.

### 2.11. Cell Binding Analysis

The binding kinetics of [^99m^Tc]Tc-Z_HER3_-ABD-mcDM1, [^99m^Tc]Tc-Z_HER3_-ABD-AA and [^99m^Tc]Tc-(HE)_3_-Z_HER3_ to BxPC-3 cells were measured in real-time using a LigandTracer Yellow (Ridgeview Instruments, Uppsala, Sweden). Cells were plated in a dedicated area of a 10 cm petri dish 1 d before the experiment. The petri dish was placed in a rotating holder and three concentrations of radiolabeled construct were added stepwise to the dish (0.3 nM, 1 nM, 3 nM), starting from the lowest concentration. The next concentration was added when the signal had reached equilibrium. The radioactive solution was replaced by fresh culture medium to record the dissociation from the cells. Data were analyzed and k_a_, k_d_, and K_D_ were determined by TraceDrawer Software (Version 1.9.2, Ridgeview Instruments, Uppsala, Sweden). The measurements were performed in triplicate at room temperature.

### 2.12. Animal Studies

Female BALB/c nu/nu mice were inoculated with HER3-expressing BxPC-3 (*n* = 28) or DU145 cells (*n* = 8), or HER3-negative RAMOS cells (*n* = 8) 2–3 weeks before the biodistribution experiment. At the start of the experiment, the average animal weight was 18 ± 1 g, and the average tumor weights were 0.08 ± 0.06 g (BxPC-3), 0.07 ± 0.05 g (DU145), and 0.09 ± 0.04 g (RAMOS). For each data point, a group of four animals was used.

The biodistribution of [^99m^Tc]Tc-Z_HER3_-ABD-mcDM1 and [^99m^Tc]Tc-Z_HER3_-ABD-AA was studied in BxPC-3 xenografted mice, 1, 6, and 24 h pi and in DU145-xenografted mice, 24 h pi. Mice were injected intravenously with 20 µg of the affibody constructs, the injected activity was adjusted to be 40 kBq at the time of sample collection, and the protein dose was adjusted with non-labeled construct. The biodistribution of [^99m^Tc]Tc-(HE)_3_-Z_HER3_ was included for comparison. For this control, one group of BxPC-3-xenografted mice were injected with 2 μg (40 kBq) of [^99m^Tc]Tc-(HE)_3_-Z_HER3_ and were sacrificed 1 h pi. All animals were sacrificed by ip injection of a ketamine (250 mg/kg) and xylazine (25 mg/kg) solution followed by heart puncture. Samples from the blood, salivary gland, lung, liver, spleen, stomach (without content), small intestine (without content), kidney, tumor, muscle, and bone were collected, weighed, and measured in an automated gamma counter. The gastrointestinal tract and carcass were collected and measured for activity content.

For the in vivo specificity test, mice with HER3-negative RAMOS xenografts were injected with 20 µg (640 kBq) of [^99m^Tc]Tc-Z_HER3_-ABD-mcDM1 or [^99m^Tc]Tc-Z_HER3_-ABD-AA and were sacrificed 24 h pi according to the protocol described above.

## 3. Results

### 3.1. Drug Conjugate Design

**Figure 1:**
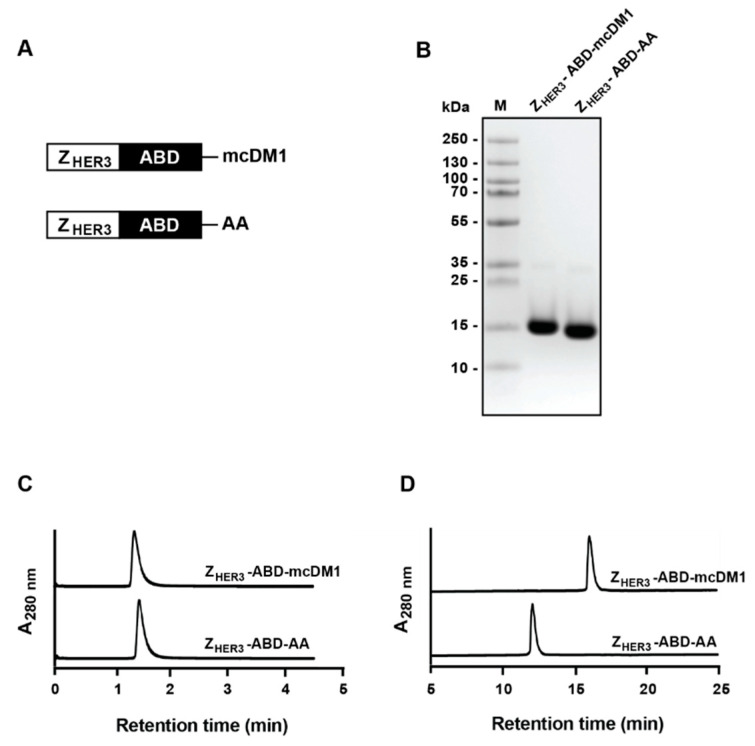
Schematic description and biochemical characterization of the drug conjugate Z_HER3_-ABD-mcDM1 and the non-toxic control Z_HER3_-ABD-AA. (**A**) The schematic figure shows the two constructs, Z_HER3_-ABD-mcDM1 and Z_HER3_-ABD-AA. (**B**) Samples (10 μg each) of the two constructs were analyzed by sodium dodecyl sulfate polyacrylamide gel electrophoresis (SDS-PAGE) under reducing conditions. Lane M corresponds to the electrophoretic separation of marker proteins, with the indicated molecular weights, 10 to 250 kDa, to the left of the gel. (**C**) Size-exclusion chromatography analysis of the two constructs. (**D**) RP-HPLC analysis of Z_HER3_-ABD-mcDM1 and Z_HER3_-ABD-AA. The constructs were eluted with a 25 min linear gradient from 30% to 60% acetonitrile in water supplemented with 0.1% trifluoroacetic acid.

To study the potential of targeting HER3-expressing tumors with an affibody-based drug conjugate, Z_HER3_-ABD-mcDM1 was generated. It consisted of Z_HER3_, an affibody molecule with a strong and specific affinity for HER3 linked via the C-terminus to an albumin-binding domain (ABD) for in vivo half-life extension. Flanking the fusion protein was an N-terminal tag for radiolabeling with [^99m^Tc]Tc, with the amino acid sequence, His-Glu-His-Glu-His-Glu and a C-terminal ending of Glu-Glu-Glu-Cys. The cysteine was used to site-specifically attach DM1 via a non-cleavable malemidocaproyl (mc) linker (Figure 1A).

### 3.2. Protein Expression, DM1 Conjugation, and Biochemical Characterization

The fusion protein Z_HER3_-ABD was recombinantly expressed in *Escherichia coli* in a soluble form. It was purified by affinity chromatography using immobilized human serum albumin (HSA) as the ligand. After purification, mcDM1 was conjugated with the C-terminal cysteine yielding Z_HER3_-ABD-mcDM1. A non-toxic control was also created where the cysteine was alkylated, yielding Z_HER3_-ABD-AA (Figure 1A). Both constructs were purified by reversed-phase high-performance liquid chromatography (RP-HPLC). Samples of Z_HER3_-ABD-mcDM1 and Z_HER3_-ABD-AA were analyzed by SDS-PAGE (Figure 1B). According to the gel, both constructs were of essentially the correct molecular weight with no visible extra bands, indicating a high purity. The constructs were further analyzed by size-exclusion chromatography under native conditions (Figure 1C). Both constructs were eluted as single, symmetrical peaks, suggesting that they were in a monomeric state, and that no oligo- or multimers were formed. Both Z_HER3_-ABD-mcDM1 and Z_HER3_-ABD-AA were analyzed by analytical RP-HPLC and were eluted essentially as single peaks (Figure 1D). Calculation of the area under curve (AUC), showed that they were of more than 95% purity, close to 100%. In Figure 1D it is evident that Z_HER3_-ABD-mcDM1 was eluted later than the non-toxic control. This was not surprising since DM1 is relatively hydrophobic.

**Figure 2:**
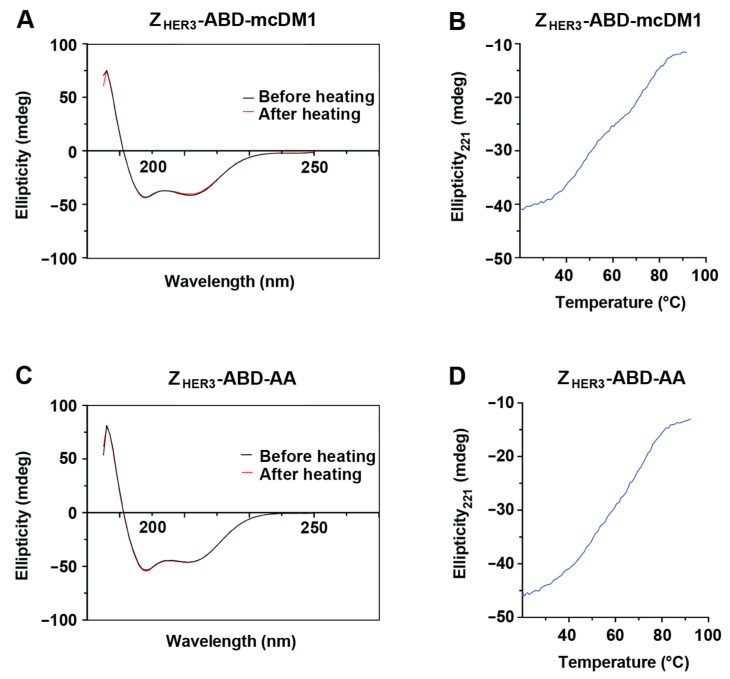
Circular dichroism measurements. Panels (**A**,**C**) show the measured ellipticity (*y*-axis) at different wavelengths (*x*-axis). In both panels the spectra obtained before and after heat-induced denaturation followed by refolding are included. The ellipticity at 221 nM was also measured as a function of temperature (Panel (**B**,**D**)). The secondary structure of the constructs is to a large extent alphahelical and the ellipticity at 221 nm is therefore a measurement of the folded state of the constructs.

The constructs were further subjected to circular dichroism analysis. The spectra of both Z_HER3_-ABD-mcDM1 and Z_HER3_-ABD-AA showed a high level of alpha-helicity, characteristic of affibody molecules and the ABD (Figure 2A,C). The thermal stability of the constructs was also investigated by measuring the ellipticity at 221 nm during heating (Figure 2B,D). At this wavelength, the highly alpha-helical constructs have a large negative ellipticity when folded. The negative ellipticity is lost upon heating, which indicates the unfolding of the conjugates. After heat-induced denaturation, the samples were cooled to 25 °C, and new spectra were recorded and overlayed with the spectra recorded before heating (Figure 2A,C). For both constructs, the spectra before and after heating were essentially identical, suggesting efficient refolding after heat-induced denaturation.

### 3.3. Determination of Affinity to HER3 and Murine ErbB3

The affinities of Z_HER3_-ABD-mcDM1 and Z_HER3_-ABD-AA to HER3 and murine ErbB3 (mErbB3) were determined by surface plasmon resonance analysis, and the results are displayed in Table 1.

The affinities of Z_HER3_-ABD-mcDM1 to HER3 and mErbB3 at 25 °C were similar with equilibrium dissociation constants (K_D_ values) of 6 nM and 5 nM, respectively. The affinity measurements were also performed at 37 °C to have a milieu closer to the in vivo situation. The affinities were weaker at this temperature, particularly to mErbB3, with K_D_ values of 12 nM (HER3) and 100 nM (mErbB3). At both 25 °C and 37 °C, the affinities obtained after heat-induced denaturation were essentially the same, further showing the efficient and functional refolding of the drug conjugate. For Z_HER3_-ABD-AA, a similar pattern was found with similar K_D_-values for HER3 and mErbB3 at 25 °C, but with a weaker affinity for the murine variant at 37 °C. Moreover, this construct had similar affinities before and after heat-induced denaturation, showing efficient refolding. Generally, the affinities measured for Z_HER3_-ABD-AA were slightly stronger than the affinities measured for Z_HER3_-ABD-mcDM1.

The affinity measurement was set up as a capture assay, where HSA was immobilized on the sensor chip, followed by the injection of a construct and then HER3 or mErbB3. The results thus show that in all cases, the constructs were able to bind to HSA and HER3 or mErbB3 simultaneously.

### 3.4. Determination of Cytotoxic Potential

**Figure 3:**
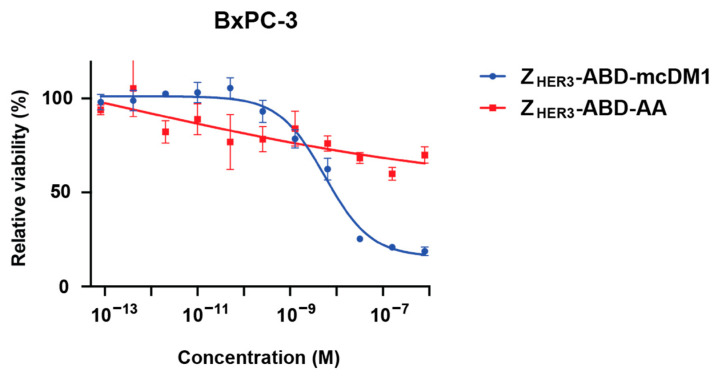
Effect on viability on the human pancreatic cancer cell line BxPC-3 by Z_HER3_-ABD-mcDM1 and Z_HER3_-ABD-AA. The cells were incubated with different concentrations of the constructs (*x*-axis), followed by the measurement of cell viability (*y*-axis). The viability of cells grown without the addition of any construct was set to 100%. Each data point is the average of four experiments. The error bars correspond to 1 SD.

An essential characteristic of drug conjugates is their ability to target the relevant cells and deliver a cytotoxic effect. To investigate the cytotoxic effect of Z_HER3_-ABD-mcDM1 to HER3-overexpressing cells, a pancreatic carcinoma model was chosen. This is one of the cancer types where HER3 overexpression has been correlated to a more dismal prognosis for the patients [6]. The cytotoxic effect was investigated by treating BxPC-3 cells with different concentrations of Z_HER3_-ABD-mcDM1, followed by the determination of cell viability (Figure 3). Z_HER3_-ABD-mcDM1 showed a dose-dependent cytotoxic effect with an IC_50_ value of 7 nM. Treatment of the cells with the non-toxic control Z_HER3_-ABD-AA showed some loss in viability at higher concentrations.

### 3.5. Radiolabeling with [^99m^Tc]Tc

To allow further analysis in vitro and in vivo, Z_HER3_-ABD-mcDM1 and Z_HER3_-ABD-AA were radiolabeled with the residualizing nuclide, [^99m^Tc]Tc. Specifically, the constructs were labeled with [^99m^Tc][Tc(CO)_3_(H_2_O)_3_]^+^ on the N-terminal (HE)_3_-tag. The radiochemical yields were 93.0 ± 2% and 90.9 ± 0.4% for [^99m^Tc]Tc-Z_HER3_-ABD-mcDM1 and [^99m^Tc]Tc-Z_HER3_-ABD-AA, respectively. After purification by size exclusion chromatography, the purity for both constructs was greater than 99.9%. A control construct to be used in vivo, (HE)_3_-Z_HER3_, was also radiolabeled and purified. The resulting purity after size-exclusion chromatography was >98%.

To test the stability of the [^99m^Tc]Tc-label, the constructs were challenged with a high concentration of histidine. The results are shown in Table 2. Only a minor release of the label, up to 2.3% for [^99m^Tc]Tc-Z_HER3_-ABD-mcDM1, was observed at room temperature at both 1 h and 4 h. The release was higher for both constructs at 37 °C with 8.8 ± 0.6%, and 5.7 ± 0.2% released within 4 h from [^99m^Tc]Tc-Z_HER3_-ABD-mcDM1 and [^99m^Tc]Tc-Z_HER3_-ABD-AA, respectively. Only a minor release was observed in the control experiment (PBS), which was performed in a PBS buffer without histidine.

### 3.6. Cell-Binding Specificity and Rate of Internalization

**Figure 4:**
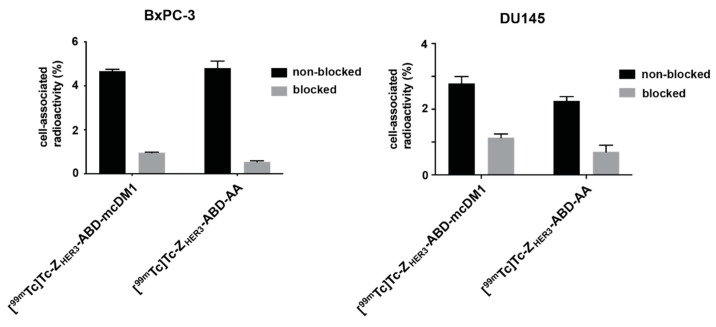
Cell-binding specificity of [^99m^Tc]Tc-Z_HER3_-ABD-mcDM1 and [^99m^Tc]Tc-Z_HER3_-ABD-AA. BxPC-3 or DU145 cells were pre-incubated (blocked) with Z_HER3:08698_ or not pre-incubated (non-blocked). The concentration of the construct for pre-blocking was 50 nM. Subsequently, the cells were incubated with [^99m^Tc]Tc-Z_HER3_-ABD-mcDM1 or [^99m^Tc]Tc-Z_HER3_-ABD-AA (0.1 nM). Both panels show the activity retained on the cells after incubation, as the percentage of the activity initially added. The results are shown as the average of three samples with the error bars corresponding to 1 SD.

The interaction of the radiolabeled constructs with the pancreatic carcinoma cell line BxPC-3 and the prostate carcinoma cell line DU145, was further characterized. The specificity in the interaction was determined by incubating the cells with [^99m^Tc]Tc-Z_HER3_-ABD-mcDM1 or [^99m^Tc]Tc-Z_HER3_-ABD-AA, and comparing the result with cells where available HER3 receptors had been pre-blocked with a large excess of the non-ABD conjugated, non-radiolabeled Z_HER3_ affibody. The experiment showed that the blocking of available HER3 receptors resulted in a significant decrease in the uptake of [^99m^Tc]Tc-Z_HER3_-ABD-mcDM1 and [^99m^Tc]Tc-Z_HER3_-ABD-AA in both cell lines (Figure 4), suggesting a HER3-specific uptake of the constructs.

**Figure 5:**
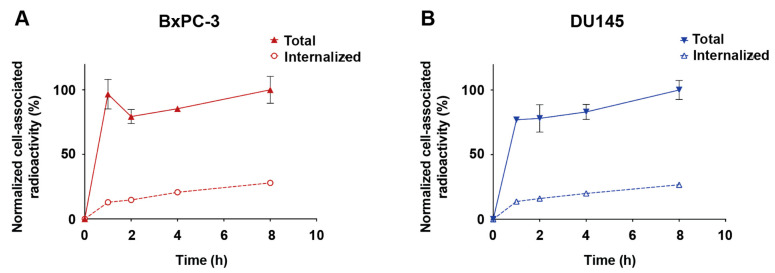
Cellular association and internalization of [^99m^Tc]Tc-Z_HER3_-ABD-mcDM1 in BxPC-3 (**A**) and DU145 (**B**) cells. The cells, BxPC-3 and DU145, were subjected to continuous incubation with 0.1 nM [^99m^Tc]Tc-Z_HER3_-mcDM1 at 37 °C. The percent of total cell-associated radioactivity and internalized radioactivity was measured and is plotted as a function of time. All values were normalized to the highest value observed (which was set to 100%). The data points represent the average values measured for three samples with error bars corresponding to 1 standard deviation. In some cases, the error bars are smaller than the symbols, and are therefore not visible.

Next, the rates of association and internalization of [^99m^Tc]Tc-Z_HER3_-ABD-mcDM1 were investigated. The association of [^99m^Tc]Tc-Z_HER3_-ABD-mcDM1 to both cell lines was quick with more than 75% of the total cell-associated activity bound after 1 h of incubation (Figure 5). The level of internalized radioactivity increased with time, and after 8 h it was 27% of the cell-associated activity for both cell lines. The difference between BxPC-3 and DU145 was not significant.

### 3.7. Affinity to BxPC-3 Cells

The affinities (equilibrium dissociation constants, K_D_ values) of [^99m^Tc]Tc-Z_HER3_-ABD-mcDM1 and [^99m^Tc]Tc-Z_HER3_-ABD-AA to HER3-expressing BxPC-3 cells were measured, and the kinetic parameters of the interactions are shown in Table 3. The K_D_ values of [^99m^Tc]Tc-Z_HER3_-ABD-mcDM1 and [^99m^Tc]Tc-Z_HER3_-ABD-AA were in the sub-nanomolar range. The control construct used in the in vivo experiments, [^99m^Tc]Tc-(HE)_3_-Z_HER3_, was also characterized. The K_D_ value of that construct for BxPC-3 cells was stronger (0.04 nM) compared to the values for [^99m^Tc]Tc-Z_HER3_-ABD-mcDM1 and [^99m^Tc]Tc-Z_HER3_-ABD-AA. The interactions of the constructs and the HER3-binding mAb seribantumab with BxPC-3 cells was also investigated by flow cytometry. Both constructs and seribantumab were found to bind to BxPC-3 cells (Figure S1).

### 3.8. Biodistribution

**Figure 6:**
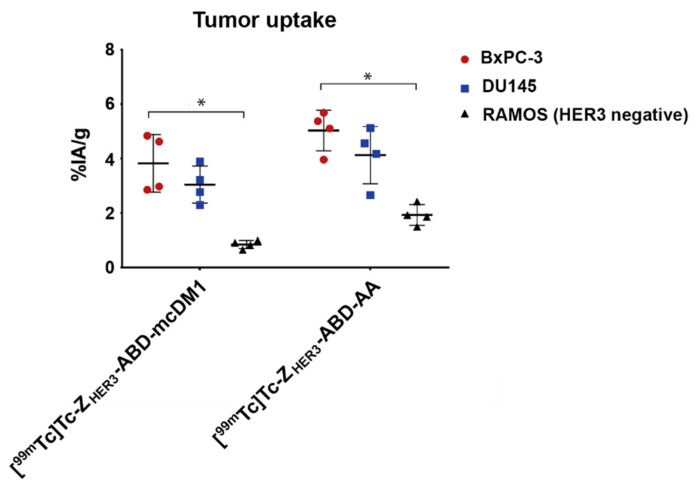
In vivo tumor targeting. The uptake (expressed as %IA/g) of [^99m^Tc]Tc-Z_HER3_-ABD-mcDM1 and [^99m^Tc]Tc-Z_HER3_-ABD-AA was studied in mice carrying HER3-expressing BxPC-3 or DU145-derived xenografts and in HER3-negative RAMOS-derived xenografts at 24 h pi. The mice (*n* = 4 animals/group) were injected with 20 µg of [^99m^Tc]Tc-labeled constructs. * corresponds to significant differences (*p <* 0.05).

The biodistribution of [^99m^Tc]Tc-Z_HER3_-ABD-mcDM1 and [^99m^Tc]Tc-Z_HER3_-ABD-AA was studied in animals with xenografts derived from the BxPC-3 or DU145 cell lines, both expressing HER3. As a control, xenografts derived from the RAMOS cell line were used, which do not express HER3. The uptake of [^99m^Tc]Tc-Z_HER3_-ABD-mcDM1 and [^99m^Tc]Tc-Z_HER3_-ABD-AA was significantly higher in the BxPC-3 xenografts than in the RAMOS-derived xenografts at 24 h, indicating a specific binding of the constructs to HER3 in vivo (Figure 6). The uptake in the four DU145 xenografts at 24 h was higher than the uptake in the four RAMOS xenografts, but the difference was not significant. Furthermore, there was no significant difference in tumor uptake between [^99m^Tc]Tc-Z_HER3_-ABD-mcDM1 and [^99m^Tc]Tc-Z_HER3_-ABD-AA.

**Figure 7:**
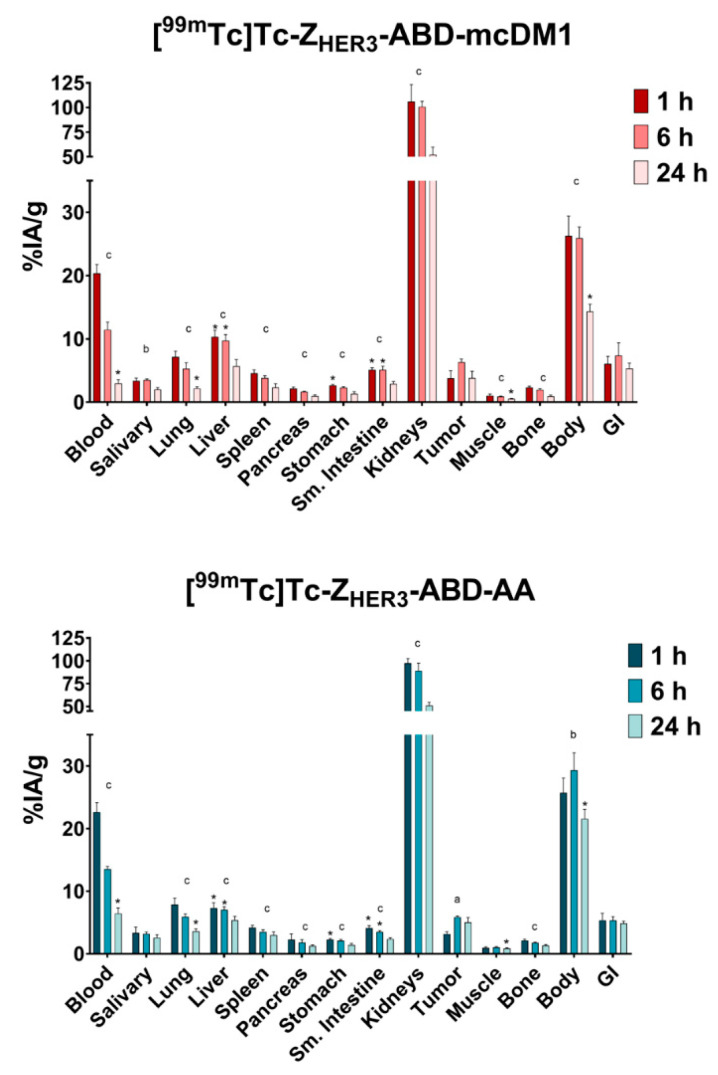
General biodistribution of [^99m^Tc]Tc-Z_HER3_-ABD-mcDM1 and [^99m^Tc]Tc-Z_HER3_-ABD-AA. Analyses were performed at 1 h, 6 h, and 24 h pi in mice bearing BxPC-3-derived xenografts. The bars represent the average of four mice. Data are presented as the uptake in different organs as a percentage of the injected activity divided by the weight of the organ or tumor (%IA/g). The data for the GI tract and body are presented as %IA per whole sample. Each mouse received an injection of 20 µg of the [^99m^Tc]Tc-labeled conjugates. * corresponds to statistically significant differences (*p <* 0.05) between [^99m^Tc]Tc-Z_HER3_-ABD-mcDM1 and [^99m^Tc]Tc-Z_HER3_-ABD-AA for the same organ at the same time-point. ^a^ corresponds to significant differences (*p <* 0.05) between the 1 and 6 h time points. ^b^ corresponds to significant differences (*p <* 0.05) between the 6 h and 24 h time points. ^c^ corresponds to significant differences (*p <* 0.05) between the 1 h and 24 h time points.

The general biodistribution of [^99m^Tc]Tc-Z_HER3_-ABD-mcDM1 and [^99m^Tc]Tc-Z_HER3_-ABD-AA in mice carrying BxPC-3 xenografts was also studied as a function of time (Figure 7, Table S1). The results demonstrated an uptake in organs with the expression of mErbB3 (liver, lung, salivary gland, stomach, and small intestine) and an additionally elevated uptake in the spleen. Considering all organs and the tumors, the uptake of [^99m^Tc]Tc-Z_HER3_-ABD-mcDM1 was significantly lower at 24 h pi compared with the uptake at 1 h pi with the exceptions of the salivary gland, tumor, and GI tract. A similar pattern was observed for animals injected with [^99m^Tc]Tc-Z_HER3_-ABD-AA with the exception of salivary gland, tumor, muscle, and GI tract. A comparison of the uptake of [^99m^Tc]Tc-Z_HER3_-ABD-mcDM1 and [^99m^Tc]Tc-Z_HER3_-ABD-AA showed some differences. At both 1 h and 6 h pi, the uptake in the liver of [^99m^Tc]Tc-Z_HER3_-ABD-mcDM1 was significantly higher compared with the hepatic uptake of [^99m^Tc]Tc-Z_HER3_-ABD-AA. Moreover, at 1 and 6 h, the uptake of [^99m^Tc]Tc-Z_HER3_-ABD-mcDM1 in the small intestine was higher than the uptake of [^99m^Tc]Tc-Z_HER3_-ABD-AA. For the tumors, the uptake increased significantly from 1 h to 6 h pi, where it peaked with 6.3 ± 0.4% IA/g and 5.9 ± 0.2% IA/g, for [^99m^Tc]Tc-Z_HER3_-ABD-mcDM1 and [^99m^Tc]Tc-Z_HER3_-ABD-AA, respectively.

As a control, the biodistribution of [^99m^Tc]Tc-(HE)_3_-Z_HER3_ was studied at 1 h pi (Table S2) This construct lacked both the half-life extending ABD domain, and the cytotoxic DM1 molecule. The uptake of [^99m^Tc]Tc-(HE)_3_-Z_HER3_ was significantly lower than the uptake of both [^99m^Tc]Tc-Z_HER3_-ABD-mcDM1 and [^99m^Tc]Tc-Z_HER3_-ABD-AA in most organs, with the exception of the kidneys where the uptake was significantly higher. Compared with [^99m^Tc]Tc-(HE)_3_-Z_HER3_, both [^99m^Tc]Tc-Z_HER3_-ABD-mcDM1 and [^99m^Tc]Tc-Z_HER3_-ABD-AA had a significant, 10-fold, higher concentration in blood at 1 h pi, showing the impact of including the ABD in the constructs for an increased in vivo half-life. For both ABD-containing constructs, a significant decrease in activity in the blood was observed between the 1 h and 24 h time points. At 24 h the blood concentration of [^99m^Tc]Tc-Z_HER3_-ABD-mcDM1 was comparable to the concentration of [^99m^Tc]Tc-(HE)_3_-Z_HER3_ at 1 h pi, and was significantly lower than the blood concentration of [^99m^Tc]Tc-Z_HER3_-ABD-AA, showing a more rapid clearance of the construct with DM1.

Furthermore, the biodistribution of [^99m^Tc]Tc-Z_HER3_-ABD-mcDM1 and [^99m^Tc]Tc-Z_HER3_-ABD-AA was studied in mice carrying DU145-derived xenografts at 24 h pi (Table S3). There was no significant difference in tumor uptake compared to the BxPC-3 model. Moreover, as expected, no difference in general biodistribution in normal organs was observed between the two xenografts models.

## 4. Discussion

Targeted therapy directed to the human epidermal growth factor receptor 3 (HER3) appears challenging due to its expression on several normal organs and relatively low over-expression on tumor cells [18]. HER3-targeted therapies have been investigated in both clinical and pre-clinical settings in several tumor types, but so far, no drugs have been approved for clinical use. It is not clear if any of the currently investigated modalities will reach the clinic and there is thus a need to also investigate other drug formats.

Here, a novel type of HER3-targeted drug candidate was constructed and studied, Z_HER3_-ABD-mcDM1, an affibody–drug conjugate (AffiDC). It is a well-defined and relatively small compound (Mw 14 kDa), consisting of a HER3-targeting affibody molecule, an albumin-binding domain for in vivo half-life extension, and the highly potent cytotoxic drug DM1. Similar to several monoclonal antibodies (mAbs) directed against HER3, Z_HER3_ by itself has been found to be able to block the activation of the receptor by HRG. In an earlier study by our group, experimental therapy with the Z_HER3_-ABD fusion protein led to a cytostatic effect, delayed tumor growth, and prolonged survival in xenograft-bearing mice, comparable to the therapeutic effect of the mAb seribantumab [43]. In the present study, we found a decreased viability of the HER3-expressing pancreatic cancer cell line BxPC-3 in the presence of increasing concentrations on the non-toxic control construct Z_HER3_-ABD-AA (Figure 3), which may have been the result of the cytostatic effect observed earlier. However, from comparing the effect of Z_HER3_-ABD-mcDM1 and Z_HER3_-ABD-AA in Figure 3, it was evident that the main inhibitory effect on cell viability was delivered through the cytotoxic drug DM1. It can therefore be concluded that Z_HER3_-ABD-mcDM1 has two modes of action, blocking the binding site of HRG and poisoning by DM1.

Monoclonal antibodies under pre-clinical and clinical development targeting HER3 usually deliver an antibody-dependent cell-mediated cytotoxicity (ADCC) effect, sometimes coupled with a blocking of the HRG-binding site and/or locking of the receptor in a conformation that prevents its dimerization and signaling, and/or triggering internalization of HER3. A more recent strategy for HER3-targeted cancer therapy, which inspired us to investigate the AffiDCs, is the antibody–drug conjugates (ADCs). These compounds take advantage of the targeting ability of the mAb, as well as the therapeutic effects described above for mAbs, and add the cytotoxic action of the attached drug. There is currently one HER3-targeting ADC in clinical development, patritumab deruxtecan, and it has shown promising results in a phase I/II trial [15]. Compared to patritumab deruxtecan, Z_HER3_-ABD-mcDM1 is considerably smaller, and should thus penetrate solid tumors more efficiently, possibly resulting in more efficient therapy [44,45]. These two molecules also contain drugs with different toxic effects; patritumab deruxtecan inhibits topoisomerase 1 activity, and Z_HER3_-ABD-mcDM1 prevents tubulin polymerization. Therefore, it is possible that the two compounds could be used concomitantly for a synergistic therapeutic effect with limited toxicity to healthy tissue, since they likely have differences in their general biodistributions.

Engineered alternative scaffold proteins (ESPs) have been gaining attention as delivery vehicles for different payloads, including cytotoxic drugs and radionuclides [20,46,47]. In addition to their smaller size, leading to a higher rate of tumor penetration [48], the use of affibody molecules also allows for production in procaryotic hosts, resulting in a comparatively low cost-of-goods compared to more advanced host cells [49]. Affibody molecules have even been produced by peptide synthesis [50], which may further reduce the cost in an industrial-scale manufacturing process.

During the biochemical evaluation of our novel AffiDC, the equilibrium dissociation constants (K_D_ values) for HER3 and murine ErbB3 were evaluated in a Biacore biosensor and were found to differ by 10-fold at 37 °C, 11 nM, and 100 nM, respectively. It is therefore possible that the on-target uptake in normal organs will be higher for a human subject than for the mice investigated in this study. However, the conditions during the experiment could only mimic the in vivo conditions. It is also interesting to note that the K_D_ values obtained at 25 °C for the two receptors were similar, with 6 nM and 5 nM for the human and murine version, respectively. The results highlight the importance of performing experiments as close to the relevant temperature as possible, 37 °C in this case.

Furthermore, the affinity of Z_HER3_-ABD-mcDM1 to the HER3-expressing BxPC-3 cells was stronger (K_D_ 0.2 nM) than the measured affinity to the human receptor with the biosensor (6 nM). It highlights the importance of studying interactions with several methods to be able to draw robust conclusions about their performance. A comparison of the affinity of Z_HER3_-ABD-mcDM1 and the non-toxic control Z_HER3_-ABD-AA to BxPC-3 cells showed the same strong affinity (K_D_ 0.2 nM) in both cases. This strongly suggests that the addition of DM1 to the C-terminus of the affibody carrier does not affect its binding properties to HER3. This finding was corroborated by the biodistribution experiment where the uptake of both constructs by the BxPC-3 and DU145 xenografts was similar.

The biodistribution of [^99m^Tc]Tc-Z_HER3_-ABD-mcDM1 and [^99m^Tc]Tc-Z_HER3_-ABD-AA was studied in tumor-bearing mice, with the measurement of the uptake in organs and tissues as a function of time. Since HER3 is expressed on some normal organs and tissues, it is important to note that Z_HER3_ is cross-reactive, and thus allows for an investigation of both the on-target and off-target uptake, to be able to determine if future pre-clinical and clinical testing is feasible. The results showed a striking difference in uptake by HER3-positive and negative tumors. The uptake of [^99m^Tc]Tc-Z_HER3_-ABD-mcDM1 was 10-fold higher in the HER3-positive xenografts derived from BxPC-3 cells, compared to the HER3-negative xenografts derived from RAMOS cells. Moreover, the relatively high kidney uptake of [^99m^Tc]Tc-Z_HER3_-ABD-mcDM1, in combination with a relatively low uptake in the liver suggests elimination mainly by the kidneys. The biodistribution pattern for [^99m^Tc]Tc-Z_HER3_-ABD-mcDM1 over time showed a significant decrease in uptake in all normal organs, which in general terms, followed the activity concentration in blood (Figure 7). There was a notable delay in the excretion of the activity for the kidney and liver that could be explained by the degradation of the labeled construct and the elimination of radiocatabolites in these organs, since the [^99m^Tc]Tc(CO)_3_ label had residualizing properties. This is in agreement with the constant activity uptake in the GI tract that was collected together with its content, and overall reflects hepatobiliary excretion to some extent. For the tumors, the activity instead accumulated over time, suggesting binding followed by internalization. Such a pattern of tumor activity uptake is in a good agreement with the in vitro data on the association and internalization of the constructs (Figure 5).

The uptake in the liver of [^99m^Tc]Tc-Z_HER3_-ABD-mcDM1 was significantly higher than the uptake of [^99m^Tc]Tc-Z_HER3_-ABD-AA, at 1 h and 6 h, possibly due to the hydrophobic character of the DM1 payload. An earlier study on an affibody–drug conjugate targeting HER2 showed that the addition of three hydrophilic and negatively charged glutamic acids next to the cysteine where DM1 was attached efficiently lowered liver accumulation [39]. In the present study, three glutamic acid residues were added in the same way to Z_HER3_-ABD-mcDM1. It is possible that the uptake of Z_HER3_-ABD-mcDM1 in the liver would have been higher without the glutamic acid residues.

Affibody-based drug conjugates targeting the HER2 receptor have been studied [36,39,40,51]. Similar to those studies, the drug conjugate in this study was able to deliver a strong cytotoxic effect to cells overexpressing the receptor, with an IC_50_ value of 7 nM to BxPC-3 cells. However, it was evident from the study on Z_HER2_-ABD-mcDM1 that the cytotoxic potential varied among different cell lines, all having a very high expression level of HER2 (AU565, SKBR3 and SKOV3), and that the strength of cell binding can only be partially correlated to the cytotoxic effect. The IC_50_ value of Z_HER2_-ABD-mcDM1 towards SKOV-3 cells was 33 nM, which was weaker than the IC_50_ value of Z_HER3_-ABD-mcDM1 towards BxPC-3 cells (7 nM). Since Z_HER2_-ABD-mcDM1 could efficiently eradicate SKOV3-derived xenografts in mice, it is likely that Z_HER3_-ABD-mcDM1 might be effective in future pre-clinical development, if the animals can tolerate a similar dosing scheme. The liver is often a sensitive organ and may limit the maximum tolerated dose. Both constructs were taken up in the liver to a relatively low extent, with 6% IA/g, 4 h after injection of 6 μg Z_HER2_-ABD-mcDM1, compared to 9.7% IA/g, 6 h after the injection of 20 μg Z_HER3_-ABD-mcDM1 (Figure 7, Table S1). The uptake in the tumors was similar with 4.2% IA/g and 6.3% IA/g for Z_HER2_-ABD-mcDM1 and Z_HER3_-ABD-mcDM1, respectively.

In conclusion, we generated a HER3-targeting affibody-based drug conjugate, Z_HER3_-ABD-mcDM1, and extensively investigated its in vitro properties as well as determined its biodistribution in tumor-bearing mice. The conjugate is a well-defined and robust molecule, with potent cytotoxicity to HER3-overexpressing cells in vitro and with the ability to target HER3-expressing human tumors in mice. The results hold promise for further pre-clinical and clinical development.

## Figures and Tables

**Table 1:** Affinity between the constructs and HER3 and mErbB3, analyzed by a real-time biosensor.

Ligand	Temperature (°C)	Analyte	k_a_ (1/Ms)	k_d_ (1/s)	K_D_ (nM)
Z_HER3_-ABD-mcDM1	25	HER3	4.3 × 10^4^	2.5 × 10^−4^	6
Z_HER3_-ABD-AA	25	HER3	1.4 × 10^5^	2.5 × 10^−4^	2
Z_HER3_-ABD-mcDM1	25	mErbB3	1.1 × 10^5^	5.7 × 10^−4^	5
Z_HER3_-ABD-AA	25	mErbB3	1.0 × 10^5^	4.7 × 10^−4^	5
Z_HER3_-ABD-mcDM1 *	25	HER3	6.0 × 10^4^	2.2 × 10^−4^	4
Z_HER3_-ABD-AA *	25	HER3	1.5 × 10^5^	2.3 × 10^−4^	2
Z_HER3_-ABD-mcDM1 *	25	mErbB3	5.6 × 10^4^	4.3 × 10^−4^	8
Z_HER3_-ABD-AA *	25	mErbB3	1.0 × 10^5^	4.7 × 10^−4^	5
Z_HER3_-ABD-mcDM1	37	HER3	1.3 × 10^5^	1.5 × 10^−3^	10
Z_HER3_-ABD-AA	37	HER3	2.2 × 10^5^	1.2 × 10^−3^	5
Z_HER3_-ABD-mcDM1	37	mErbB3	2.7 × 10^4^	2.7 × 10^−3^	100
Z_HER3_-ABD-AA	37	mErbB3	1.2 × 10^5^	2.7 × 10^−3^	20
Z_HER3_-ABD-mcDM1 *	37	HER3	1.6 × 10^5^	1.8 × 10^−3^	10
Z_HER3_-ABD-AA *	37	HER3	2.4 × 10^5^	1.2 × 10^−3^	5
Z_HER3_-ABD-mcDM1 *	37	mErbB3	6.6 × 10^4^	3.7 × 10^−3^	60
Z_HER3_-ABD-AA *	37	mErbB3	9.2 × 10^4^	2.4 × 10^−3^	30

* Measurement after heat-induced unfolding and refolding.

**Table 2:** Radiolabel stability of [^99m^Tc]Tc-Z_HER3_-ABD-mcDM1 and [^99m^Tc]Tc-Z_HER3_-ABD-AA.

Condition	[^99m^Tc]Tc-Z_HER3_-ABD-mcDM1		[^99m^Tc]Tc-Z_HER3_-ABD-AA	
	1 h	4 h	1 h	4 h
500× histidine, 22 °C	1.4 ± 0.5 *	2.3 ± 0.1	1.3 ± 0.1	2.0 ± 0.4
500× histidine, 37 °C	4.0 ± 0.5	8.8 ± 0.6	4.3 ± 0.5	5.7 ± 0.2
PBS, 22 °C	1.3 ± 0.9	0.9 ± 0.5	1.0 ± 0.6	1.0 ± 0.5

* The values in the table are given as a percent of the released radioactivity from the constructs ± 1 SD. Each value corresponds to the average of three replicate experiments.

**Table 3:** Kinetic parameters and equilibrium dissociation constants for the interaction between the constructs and BxPC-3 cells.

Construct	k_a_ (1/Ms) ^a^	k_d_ (1/s)	K_D_ (nM)
[^99m^Tc]Tc-Z_HER3_-ABD-mcDM1	1.4 × 10^5^ ± 0.1 × 10^5^	3.0 × 10^−5^ ± 2.0 × 10^−5^	0.2 ± 0.1
[^99m^Tc]Tc-Z_HER3_-ABD-AA	1.8 × 10^5^ ± 0.1 × 10^5^	4.0 × 10^−5^ ± 3.0 × 10^−5^	0.2 ± 0.1
[^99m^Tc]Tc-(HE)_3_-Z_HER3_	3.0 × 10^5^ ± 0.4 × 10^5^	1.1 × 10^−5^ ± 0.1 × 10^−5^	0.04 ± 0.01

^a^ Each value in the table is the average of three ([^99m^Tc]Tc-Z_HER3_-ABD-mcDM1 and [^99m^Tc]Tc-Z_HER3_-ABD-AA) or two ([^99m^Tc]Tc-(HE)_3_-Z_HER3_) independent experiments. The error corresponds to 1 standard deviation.

## Data Availability

The data generated in the study are available upon reasonable request through the corresponding authors.

## Supplementary Materials

The following supporting information can be downloaded at: https://www.mdpi.com/article/10.3390/biomedicines10061293/s1, Figure S1: Flow cytometry analysis of binding of Z_HER3_-ABD-mcDM1, Z_HER3_-ABD-AA, and seribantumab to BxPC-3 55 cells; Table S1: Biodistribution of [^99m^Tc]Tc-Z_HER3_-ABD-mcDM1, and [^99m^Tc]Tc-Z_HER3_-ABD-AA in BxPC-3 xenograft bearing mice at 1 h, 6 h, and 24 h pi; Table S2: Biodistribution of [^99m^Tc]Tc-(HE)_3_-Z_HER3_ in BxPC-3 xenograft bearing mice at 1 h pi; Table S3: Biodistribution of [^99m^Tc]Tc-Z_HER3_-ABD-mcDM1 and [^99m^Tc]Tc-Z_HER3_-ABD-AA in DU145 xenograft bearing mice at 24 h pi.
