# Supplementary material for: Targeting Tumor Cells Overexpressing the Human Epidermal Growth Factor Receptor 3 with Potent Drug Conjugates Based on Affibody Molecules
Source: Biomedicines. 2022 May 31;10(6):1293. doi: 10.3390/biomedicines10061293 (PMC9219639; doi:10.3390/biomedicines10061293)
Supplement: Supplementary file 1 [file biomedicines-10-01293-s001.zip › biomedicines-1711772-supplementary.pdf]

biomedicines

Article
Supplementary

Targeting tumor cells overexpressing the human epidermal growth factor receptor 3 with potent drug conjugates based on affibody molecules

Sara Rinne [1,†], Wen Yin [2,†], Anna Mestre Borras [2], Ayman Abouzaed [1], Charles Dahlsson Leitao [2], Anzhelika Vorobyeva [3], John Löfblom [2], Stefan Ståhl [2], Anna Orlova [1,3,4,*], and Torbjörn Gräslund [2,*]

[1] Department of Medicinal Chemistry, Uppsala University, 751 23 Uppsala, Sweden; sara.rinne@ilk.uu.se (S.R.); ayman.abouzayed@ilk.uu.se (A.A.)
[2] Department of Protein Science, KTH Royal Institute of Technology, 114 17 Stockholm, Sweden; wenyin@kth.se (W. Y.); annamb@kth.se (A.M.B.); chdl@kth.se (C.D.L.); lofblom@kth.se (J.L.); ssta@kth.se (S.S.)
[3] Department of Immunology, Genetics and Pathology, Uppsala University, 752 37 Uppsala, Sweden (A.V.)
[4] Science for Life Laboratory, Uppsala University, 752 37 Uppsala, Sweden

† These authors contributed equally to the work
* Correspondence: anna.orlova@ilk.uu.se, +46-(0)73-992 2846 (A.O.); torbjorn@kth.se, +46-(0)70-790 9627 (T.G.)

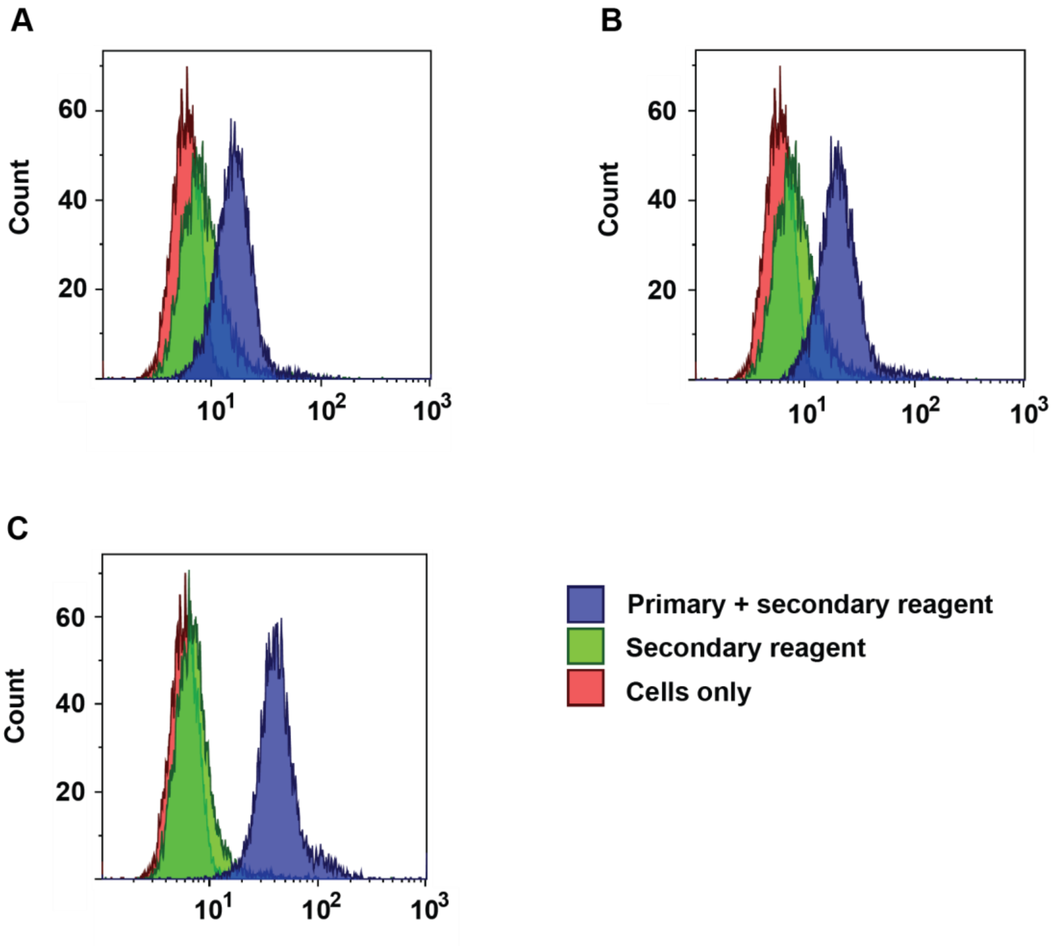

Figure S1. Flow cytometry analysis of binding of Z_{HER2}-ABD-mcDM1, Z_{HER2}-ABD-AA, and seribantumab to BxPC-3 cells. The panels show an overlay of cells stained with primary and secondary reagent, secondary reagent only, and non-stained cells. In panel (**A**), the primary reagent was Z_{HER2}-ABD-mcDM1 (100 nM) and the secondary reagent was HSA (225 nM) (Invitrogen, Waltham, MA, USA) conjugated with Alexa 647. In panel (**B**), the primary reagent was Z_{HER2}-ABD-AA (100 nM) and the secondary reagent was HSA (225 nM)(Invitrogen) conjugated with Alexa 647. In panel (**C**), the primary reagent was seribantumab (100 nM) (Atlas antibodies, Bromma, Sweden) and the secondary reagent was a goat anti-human antibody conjugated with Alexa 647 (13 nM) (Invitrogen). In all cases, BxPC-3 cells were stained with the primary reagent for 45 min at room temperature, and with secondary reagent for 30 min at 4 °C.

Table S1. Biodistribution of [^{99m}Tc]Tc-Z$_{HER3}$-ABD-mcDM1, and [^{99m}Tc]Tc-Z$_{HER3}$-ABD-AA in BxPC-3 xenograft bearing mice at 1 h, 6 h, and 24 h pi. The uptake in different organs were quantified and are presented as percent of the injected activity divided by the weight of the organ (%IA/g). Each value is the mean of four mice ± SD.

Organ	[^{99m}Tc]Tc-Z$_{HER3}$-ABD-mcDM1			[^{99m}Tc]Tc-Z$_{HER3}$-ABD-AA		
	**1 h**	**6 h**	**24 h**	**1 h**	**6 h**	**24 h**
Blood	20 ± 1	11 ± 1	3.0 ± 0.6	23 ± 2	14 ± 0.4	6.4 ± 0.9
Salivary gland	3.4 ± 0.4	3.5 ± 0.1	2.0 ± 0.3	3.3 ± 1.0	3.2 ± 0.3	2.6 ± 0.5
Lung	7.2 ± 0.9	5.3 ± 0.9	2.1 ± 0.3	7.9 ± 1.0	5.9 ± 0.5	3.6 ±0.4
Liver	10 ± 1	9.7 ±0.9	5.7 ± 1.0	7.3 ± 0.8	7.0 ± 0.5	5.4 ± 0.6
Spleen	4.6 ± 0.5	3.8 ± 0.4	2.3 ± 0.6	4.2 ± 0.4	3.5 ± 0.3	3.0 ± 0.5
Pancreas	2.1 ± 0.2	1.6 ± 0.1	1.0 ± 0.2	2.3 ± 0.9	1.8 ± 0.4	1.3 ± 0.2
Stomach	2.7 ± 0.1	2.3 ± 0.1	1.3 ± 0.3	2.3 ± 0.2	2.1 ± 0.2	1.4 ± 0.2
Small Intestine	5.1 ± 0.3	5.1 ± 0.6	2.9 ± 0.4	4.1 ± 0.4	3.5 ± 0.2	2.4 ± 0.2
Kidney	110 ± 20	100 ± 10	52 ± 8	98 ± 5	89 ± 8	51 ± 3
Tumor	3.8 ± 1.2	6.3 ± 0.5	3.8 ± 1.1	3.2 ± 0.3	5.9 ± 0.2	5.0 ± 0.8
Muscle	1.0 ± 0.3	0.87 ± 0.08	0.5 ± 0.1	0.9 ± 0.2	1.0 ± 0.1	0.9 ± 0.1
Bone	2.3 ± 0.2	2.0 ± 0.1	0.92 ± 0.21	2.1 ± 0.2	1.8 ± 0.1	1.3 ± 0.1
Body*	26 ± 3	26 ± 2	14 ± 1	26 ± 2	29 ± 3	22 ± 2
GI-tract*	6.1 ± 1.2	7.4 ± 2.0	5.3 ± 0.8	5.4 ± 1.1	5.3 ± 0.6	4.9 ± 0.3

*Data for Body and GI-tract is presented as %IA per whole sample.

Table S2. Biodistribution of [^{99m}Tc]Tc-(HE)$_3$-Z$_{HER3}$ in BxPC-3 xenograft bearing mice at 1 h pi. Mice were injected with 2 µg of [^{99m}Tc]Tc-(HE)$_3$-Z$_{HER3}$. The uptake in different organs were quantified and the values are presented as percent of the injected activity divided by the weight of the organ (%IA/g). Each value is the mean of four mice ± SD.

**Organ**	**Uptake (%IA/g)**
Blood	2.5 ± 0.1
Salivary gland	1.2 ± 0.1
Lung	2.1 ± 0.2
Liver	4.1 ± 0.1
Spleen	0.94 ± 0.03
Pancreas	0.66 ± 0.05
Stomach	1.4 ± 0.1
Small Intestine	2.6 ± 0.2
Kidney	190 ± 20
Tumor	2.9 ± 0.7
Muscle	0.35 ± 0.07
Bone	0.67 ± 0.08
Body*	11 ± 1
GI-tract*	5.5 ± 0.4

*Data for Body and GI-tract is presented as %IA per whole sample.

Table S3: Biodistribution of [^{99m}Tc]Tc-Z$_{HER3}$-ABD-mcDM1 and [^{99m}Tc]Tc-Z$_{HER3}$-ABD-AA in DU145 xenograft bearing mice at 24 h pi. Mice were injected with 20 µg of the respective radioconjugates. The uptake in different organs were quantified and the values are presented as percent of the injected activity divided by the weight of the organ (%IA/g). Each value is the mean of four mice ± SD.

Organ	[^{99m}Tc]Tc-Z$_{HER3}$-ABD-mcDM1	[^{99m}Tc]Tc-Z$_{HER3}$-ABD-AA
Blood	2.7 ± 0.4[a]	7.0 ± 1.0
Salivary	2.4 ± 0.3	2.7 ± 0.4
Lung	2.1 ± 0.3[a]	4.0 ± 0.4
Liver	5.8 ±0.8[a]	4.2 ± 0.9
Spleen	2.3 ± 0.1[a]	3.5 ± 0.8
Pancreas	1.0 ± 0.1[a]	1.4 ± 0.2
Stomach	1.6 ± 0.2	1.7 ± 0.3
Small Intestine	3.2 ± 0.5[a]	2.0 ± 0.2
Kidneys	52 ± 6	62 ± 7
Tumor	3.0 ± 0.6	4.1 ± 0.9
Muscle	0.6 ± 0.1[a]	0.8 ± 0.1
Bone	1.0 ± 0.2[a]	1.5 ± 0.2
Body*	14 ± 1[a]	19 ± 2
GI*	5.8 ± 0.4[a]	3.1 ± 0.3

[*]Data for Body and GI-tract is presented as %IA per whole sample.
[a]Statistically significant difference in uptake between [^{99m}Tc]Tc-Z$_{HER3}$-ABD-mcDM1 and [^{99m}Tc]Tc-Z$_{HER3}$-ABD-AA
